# Metabolome-wide Mendelian randomization for age at menarche and age at natural menopause

**DOI:** 10.1186/s13073-024-01322-7

**Published:** 2024-05-28

**Authors:** Mojgan Yazdanpanah, Nahid Yazdanpanah, Isabel Gamache, Ken Ong, John R. B. Perry, Despoina Manousaki

**Affiliations:** 1 Research Center of the Sainte-Justine University Hospital, Université de Montréal, 3175 Côte-Sainte-Catherine, Montréal, Québec, H3T 1C5 Canada; 2 MRC Epidemiology Unit, School of Clinical Medicine, Wellcome-MRC Institute of Metabolic Science, University of Cambridge, Cambridge, CB2 0QQ UK; 3 Metabolic Research Laboratory, School of Clinical Medicine, Wellcome-MRC Institute of Metabolic Science, University of Cambridge, Cambridge, CB2 0QQ UK; 4 Departments of Pediatrics, Biochemistry and Molecular Medicine, Université de Montréal, Montreal, Canada

**Keywords:** Metabolites, Menarche, Menopause, Mendelian randomization, ALSPAC

## Abstract

**Background:**

The role of metabolism in the variation of age at menarche (AAM) and age at natural menopause (ANM) in the female population is not entirely known. We aimed to investigate the causal role of circulating metabolites in AAM and ANM using Mendelian randomization (MR).

**Methods:**

We combined MR with genetic colocalization to investigate potential causal associations between 658 metabolites and AAM and between 684 metabolites and ANM. We extracted genetic instruments for our exposures from four genome-wide association studies (GWAS) on circulating metabolites and queried the effects of these variants on the outcomes in two large GWAS from the ReproGen consortium. Additionally, we assessed the mediating role of the body mass index (BMI) in these associations, identified metabolic pathways implicated in AAM and ANM, and sought validation for selected metabolites in the Avon Longitudinal Study of Parents and Children (ALSPAC).

**Results:**

Our analysis identified 10 candidate metabolites for AAM, but none of them colocalized with AAM. For ANM, 76 metabolites were prioritized (FDR-adjusted MR *P*-value ≤ 0.05), with 17 colocalizing, primarily in the glycerophosphocholines class, including the omega-3 fatty acid and phosphatidylcholine (PC) categories. Pathway analyses and validation in ALSPAC mothers also highlighted the role of omega and polyunsaturated fatty acids levels in delaying age at menopause.

**Conclusions:**

Our study suggests that metabolites from the glycerophosphocholine and fatty acid families play a causal role in the timing of both menarche and menopause. This underscores the significance of specific metabolic pathways in the biology of female reproductive longevity.

**Supplementary Information:**

The online version contains supplementary material available at 10.1186/s13073-024-01322-7.

## Background

Female reproductive longevity, defined by the timing of menarche and menopause, exhibits significant variability driven by genetics, lifestyle, and environmental exposures [1, 2], but the precise biological mechanisms underlying variations in reproductive aging are still not fully understood. However, the timing of both age at menarche (AAM) and age at natural menopause (ANM) appears to have significant effects on women’s health [3]. For example, the early onset of puberty has been linked to high risk-taking behaviors, reduced educational attainment [3], adult obesity, type 2 diabetes [4], cardiovascular diseases [5], susceptibility to cancers, and increased mortality rates [6]. Interestingly, women are more likely to experience an early natural menopause following either early or late menarche [7]. Therefore, identifying biomarkers that enhance our comprehension of the physiology of AAM and ANM variations, as well as their interconnectedness, is important. Moreover, these molecules may potentially serve as pharmacological targets to alter the duration of a woman’s reproductive lifespan.

Observational studies using large-scale metabolomics data have led to the discovery of a number of candidate biomarkers for various traits. Nevertheless, conducting case–control studies that simultaneously measure hundreds of circulating metabolites is cost-prohibitive but also susceptible to confounding and reverse causation, which restricts their ability to identify causal biomarkers. In recent years, large genome-wide association studies (GWAS) have identified genetic variants associated with the levels of numerous metabolites. Furthermore, large-scale GWAS datasets have become available for AAM and ANM, significantly advancing our knowledge of the genetic factors encompassing these traits. The availability of such GWAS data offers a valuable opportunity to investigate potential causal associations between circulating metabolites and AAM and ANM using Mendelian randomization (MR). MR is a well-established method in genetic epidemiology that explores whether a modifiable exposure is causally linked to a particular outcome [8]. Based on the use of genetic variants, randomly allocated at conception, to infer levels of these exposures, MR helps eliminate bias from confounding or reverse causation [9]. Two-sample MR uses data from separate GWAS for the exposure and outcome, enhancing statistical power for causal inference in complex health outcomes measured in large GWAS [10].

In this study, we conducted two-sample MR to investigate potential causal associations between hundreds of previously measured circulating metabolites and AAM or ANM using summary statistics from large GWAS [11, 12]. We further explored the potential effects of body mass index (BMI) on the MR associations between the candidate metabolites and AAM and ANM. Colocalization analyses were conducted to differentiate between causal associations and genetic correlations due to variants in linkage disequilibrium (LD). Pathway and enrichment analyses were used to uncover potential biological processes influencing AAM and ANM. Finally, we sought validation for the causal associations with AAM and age at menopause for selected candidate metabolites directly measured in participants in the Avon Longitudinal Study of Parents and Children (ALSPAC).

## Methods

### Mendelian randomization assumptions

Univariable two-sample MR studies were performed to explore potential causal relationships between circulating metabolites and AAM and ANM. MR relies on three core assumptions: (1) The genetic instrument (IV) must have a strong association with the exposure (relevance assumption); (2) the genetic instrument should not be linked to confounding factors that connect the exposure to outcome (independence assumption); (3) the genetic instrument should affect the outcome only through the exposure (exclusion restriction assumption). Violation of this last assumption is known as horizontal pleiotropy.

### Discovery datasets

**Fig. 1:**
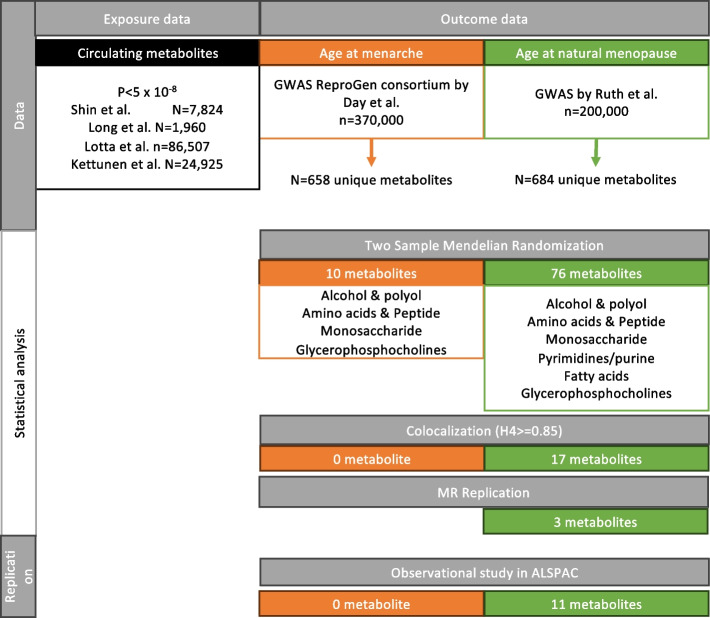
Flow chart of study design. Representation of the analytical steps and of the main results for both studied outcomes. Orange boxes refer to AAM, while green boxes refer to ANM

For our MR analysis, we collected GWAS summary statistics for circulating metabolites on Europeans to use as sources for our exposures (Kettunen et al. [13], *N* = 24,925; Lotta et al. [14], *N* = 86,507; Long et al. [15], *N* = 1960; Shin et al. [16], *N* = 7824). The samples of the GWAS by Long et al. were derived from the TwinsUK cohort, while Shin et al. performed a GWAS meta-analysis of the TwinsUK and KORA cohorts. The GWAS by Lotta et al. was a meta-analysis of four cohorts (Fenland cohort, EPIC-Norfolk, INTERVAL) while Kettunen et al. meta-analyzed 14 GWAS including two GWAS from subsets of the FINRISK97 cohort. The methods used for metabolite measurements were liquid chromatography-mass spectrometry (LC–MS) (Long et al., Lotta et al., Shin et al.), and/or gas chromatography-mass spectrometry (GS-MS) (Shin et al.), and/or nuclear magnetic resonance spectrometry (NMR) (Kettunen et al. and Lotta et al.). All GWAS adjusted their metabolite measurements for age and sex of the participants, and additional covariates appear in Additional file 1: Table S1. For the outcomes, we utilized summary statistics from the ReproGen consortium GWAS by Day et al. (*N*_total_ = 329,345, combining 40 studies with 23andMe and UK Biobank) [11] for AAM and from the largest-scale GWAS meta-analysis by Ruth et al. of four studies (1000 Genomes imputed studies, Breast Cancer Association Consortium, and UK Biobank, *N*_total_ = 201,323) [12] for ANM. Units of measurement for the exposures (metabolite levels) were standard deviations (SD), while the outcomes were expressed in years in the respective GWAS. Additional file 1: Table S1 provides additional details on each GWAS and Fig. 1 illustrates the overall study design.

### Instrumental variable selection

In order to satisfy the first MR assumption, we chose as IVs SNPs strongly associated with metabolite levels in the exposure GWAS (*P* ≤ 5 × 10^−8^). Among these, we selected independent SNPs (linkage disequilibrium (LD) *r*^2^ < 0.001) within a 500-kb region using European ancestry reference data from the 1000 Genomes Project [17]. For SNPs that were not available in the outcome GWAS, we identified proxy SNPs in high LD (*r*^2^ > 0.8) using the SNIPA website (https://snipa.helmholtz-muenchen.de/snipa3/). To further assess the first MR assumption, we filtered out metabolites for which the global *F*-statistic of the SNP-IVs was below 10, using the following formula: $F=\frac{\frac{R2}{k}}{\frac{[1-R2]}{[n-k-1]}}$, where* n* is the size of the cohort, *k* is the number of SNP-IVs, and *R*^2^ is the proportion of the variance of each exposure explained by the SNP-IVs [18] (according to the formula ${R}^{2}\approx 2{\beta }^{2}f\times \left(1-f\right)$ where $\beta$ and *f* denote the effect estimate and the effect allele frequency of the allele [19]). Summary statistics of the SNP-IVs used in our MR analysis can be found in Additional file 1: Table S2.

### Mendelian randomization analysis

We performed MR studies of the causal relationships between the exposures (metabolites) and outcomes (AAM and ANM) using the *TwoSampleMR* R package (v.0.5.5) [20]. We computed the MR Wald ratios for each genetic instrument of the exposures with the outcome, and when multiple SNP-IVs were available for a single metabolite, we meta-analyzed them using the inverse variance weighted (IVW) method [10]. Causal effects with type I error rate of less than 5% after correction for multiple testing using a false discovery rate (FDR) were considered significant.

### Sensitivity analysis

To address potential violations of the third MR assumption, we conducted several sensitivity analyses to investigate the possibility of bias introduced by genetic instruments’ heterogeneity and pleiotropy. These analyses were performed on results that met the significance threshold and required the availability of multiple SNP-IVs. To assess pleiotropy, we employed both MR-Egger regression [21] and MR-PRESSO (Pleiotropy RESidual Sum and Outlier) [22] methods. MR-Egger, unlike the IVW method, does not constrain its intercept to zero, allowing for the detection of directional pleiotropy when the intercept significantly deviates from 0 (*p*-value < 0.05). MR-Egger requires that the association of each variant with the exposure is not correlated with its pleiotropic effect, a condition known as the InSIDE assumption [21]. This is necessary to weaken the third assumption. The MR-PRESSO global test was also utilized to identify potential horizontal pleiotropy by estimating the presence of outlier SNP-IVs. As part of our sensitivity analyses, we applied Steiger filtering [23] to evaluate the directionality of the MR associations. This step ensured that the SNP-IVs were more strongly associated with the exposure (in this case, the metabolites) than with the outcomes (AAM and ANM). To assess heterogeneity, we implemented the Cochran *Q* heterogeneity test in both the IVW and MR-Egger analyses [24].

### Multivariable MR analyses

To test the second MR assumption (“independence” assumption), we tested whether the association between the candidate metabolites and AAM or ANM, as determined by our MR analysis, could be influenced by body mass index (BMI), a possible confounder or mediator. Indeed, BMI is known to influence both AAM and ANM [2, 25–27], and it also has an impact on certain metabolites [28]. In order to take this into account, we performed multivariable MR (MVMR) analysis. MVMR requires a larger number of genetic instruments for the exposures than the number of the exposures being tested in the model, which in this case are two: a metabolite and BMI. For these MVMR analyses, we used data from large available GWAS for childhood BMI (*n* = 39,620) [29] and adult BMI (*n* =  ~ 700,000) [30].

### Colocalization analyses

MR enables the detection of associations between two phenotypes; however, it is possible that the causal SNP for both phenotypes may not be the same. To explore this possibility, we performed a colocalization analysis to examine the potential influence of LD between the SNP-IVs for metabolites and the causal SNPs for AAM or ANM [31] on our causal MR associations. This analysis was performed using the coloc package in R [32], which computes posterior probabilities for four hypotheses: H0 (no association of the genomic locus with either trait), H1 (association with AAM or ANM but not with the metabolite level), H2 (association with the metabolite level but not with AAM or ANM), H3 (association with AAM or ANM and the metabolite level through two different causal SNPs in LD), and H4 (association with AAM or ANM and the metabolite level via one shared causal SNP). As parameters for prior probability, we used the default parameters, i.e., *p*_1_ (prior probability of the exposure having a causal variant) = 1.0 × 10^−4^, *p*_2_ (prior probability of the outcome having a causal variant) = 1.0 × 10^−4^, and *p*_12_ (prior probability of the exposure and the outcome sharing the same causal variant) = 1.0 × 10^−5^. To estimate the posterior probability H4 for each genomic locus, which indicates the presence of a single causal variant for both the metabolites and AAM or ANM, we analyzed all SNPs with a minor allele frequency (MAF) > 0.01 within 1 MB of each metabolite SNP-IV. Colocalization analyses were performed for metabolites that showed evidence of MR association with AAM or ANM, using the available full summary-level results from the GWAS by Lotta et al. [14], Shin et al. [16], and Kettunen et al. [13] (full summary-level results from Long et al. are not available). If the posterior probabilities of H4 were greater than 0.8 for at least one of the SNP-IV associated with a candidate metabolite, this metabolite was considered colocalized with AAM or ANM.

### Bidirectional MR

To test the directionality of our causal MR associations, in addition to the Steiger filtering, we performed reverse two-sample MR analyses, where AAM or ANM were the exposures and the colocalized metabolites were the outcomes. SNP-IVs for the two exposures (AAM or ANM) were extracted from the same ReproGen consortium GWAS and were strongly associated with the exposures at a GWAS *p*-value ≤ 5 × 10^−8^. The IVW method was used to evaluate the causal reverse associations, and we employed MR-Egger and two additional MR methods robust to pleiotropy, the weighted median [33], which assumes that at least half of the SNP-IVs are not pleiotropic, and the weighted mode [34], which assumes that the most common causal effect is consistent with the true causal effect.

### Replication of our MR findings

We sought to replicate the findings for metabolites displaying significant associations in our main MR analysis by extracting IVs for these candidate metabolites from an independent cohort by Suhre et al. [35]. Since there was no available independent GWAS with available summary statistics for the outcome (ANM), we used the same GWAS meta-analysis by Ruth et al. [12]. We identified significant IVs associated with the metabolites in the Suhre et al. study and searched for proxies for missing SNPs in the ANM GWAS using the LDproxy function of LDlinkR (*r*^2^ > 0.8) [36]. Similar to our discovery MR, replication MR analyses were performed using the TwoSampleMR package [20].

### Pathway and metabolite set enrichment analyses

To perform pathway and enrichment analyses based on the prioritized metabolites from our main MR and colocalization analyses, we first identified a single identifier per metabolite in the following databases: KEGG Compound [37], PubChem [38], BioCyc/HumanCyc [39], and Chemical Entities of Biological Interest (ChEBI) [40]. These databases provide the most frequently used and updated Human Metabolome Database (HMDB) identifiers in metabolomics [41, 42]. Over-representation analysis (ORA) was implemented using the hypergeometric test to evaluate whether a particular metabolite set is represented more than expected by chance within the given compound list. Statistical significance was determined when FDR-corrected *P*-values were below 0.05. To perform ORA, we initially provided a list of compound names, which was then consolidated using conventional feature selection techniques to explore biologically significant patterns. This involved identifying whether a specific metabolite set was more prominently represented in the given compound list than would be expected by chance. After accounting for multiple testing, one-tailed *P*-values were calculated. We then used the Gene Multiple Association Network Integration Algorithm (GeneMANIA) tool (http://www.genemania.org/) and Functional Mapping and Annotation of genetic associations (FUMA), a web-based tool (https://fuma.ctglab.nl), to construct a gene network to better characterize the functions of the main class of the MR-prioritized metabolites for AAM and ANM. Pathway analyses were performed using MetaboAnalyst [43], using “Enrichment Analysis” and “Joint-Pathway Analysis,” with the latter using the integration method of “Combine p values (pathway-level).” For the pathway and enrichment analyses, only metabolites which colocalized (H4 > 80%) with either AAM or ANM and who had identified metabolites (HMDB) were selected. These in silico follow-up analyses aimed to identify biologically meaningful pathways to which our candidate metabolites clustered, using quantitative metabolomic data.

### Validation of selected candidate metabolites in the Avon Longitudinal Study of Parents and Children (ALSPAC) study

To validate our findings for selected candidate metabolites associated with AAM and ANM, we tested the association of directly measured levels of these metabolites with the two traits in ALSPAC. The ALSPAC is a population-based birth cohort study, which enrolled 14,541 pregnant women resident in Avon, UK, with expected delivery dates between 1 April 1991 and 31 December 1992 [44, 45]. Of the initial pregnancies, there was a total of 13,988 children who were alive at 1 year of age. With additional phases of recruitment, the total sample size for analyses using any data collected after the age of seven is 15,447 pregnancies, resulting in 14,901 children being alive at 1 year of age. Overall, 8932 European children, among which *n* = 3919 girls, and their parents were closely monitored at regular intervals for 28 years using questionnaires and clinic-based assessments with full study details published elsewhere [46, 47].

Age at onset of menarche was assessed based on a derived variable, combining repeated reports at different visits from age 8 years to age 17 years [48]. Age at menopause was assessed using questionnaires from 14,541 mothers in a recent follow-up visit in 2020 and was self-reported in a questionary (Variable number: C3b). Only mothers who had their menopause were kept for analysis [49]. Information was collected at two visits (Focus on Mothers 1 and 2 or FOM1 and FOM2). BMI measurements were calculated based on height and weight measurements of girls at clinical visits at ages 7, 8, and 11 years based on the formula weight (kg)/height (cm)^2^ and were standardized to a mean of 0 and an SD of 1. Study data were collected and managed using REDCap electronic data capture tools hosted at the University of Bristol [50]. REDCap (Research Electronic Data Capture) is a secure, web-based software platform designed to support data capture for research studies. Missing BMI *z*-scores at age 8 years were imputed based on measurements at age 7 or 9 years. The maternal BMI was readily available as a derived variable, based on 2 clinic visits (FOM1 and FOM2).

Please note that the study website contains details of all the data that is available through a fully searchable data dictionary and variable search tool: http://www.bristol.ac.uk/alspac/researchers/our-data/

### Metabolite measurements in ALSPAC

Nonfasted peripheral blood was collected from ALSPAC participants (children and mothers) at four different follow-up visits, at ages 7 (F7 visit), 15 (TF3 visit), 16 (TF4 visit), and 24 years (F24 visit) for child participants. Samples were processed within 4 h and stored at − 80 °C [51]. Fasting and post-prandial blood samples were also collected for a subset of ALSPAC participants at the Before Breakfast Study (BBS) at age 8 years. In mothers, metabolite levels were measured at a fasting state either at the FOM1 visit (average age 48 years, range 34–64 years) or the FOM2 visit (average age 51 years, range 39–66 years). Metabolomic profiling was done using the Nightingale NMR metabolomics platform (Helsinki, Finland), and 228 metabolic traits (and their ratios) were quantified in EDTA-plasma.

We assessed the associations between metabolites and age at menarche or menopause using linear regression. Subsequently, to assess the influence of BMI on these associations, we included childhood BMI at age 8 years as a covariate for AAM and mothers’ adult BMI at FOM1 or FOM2 as a covariate for age at menopause. We also conducted these models without including mothers who experienced early menopause, defined as before the age of 45 [49].

## Results

### Causal relationships between metabolites and AAM or ANM

To evaluate the potential causal relationships between metabolites and AAM and ANM, we initially conducted univariate MR analyses, as outlined in the study design flowchart (Fig. 1). In total, we identified SNP-IVs for 658 metabolites for AAM and 684 for ANM (Additional file 1: Table S3).

**Fig. 2:**
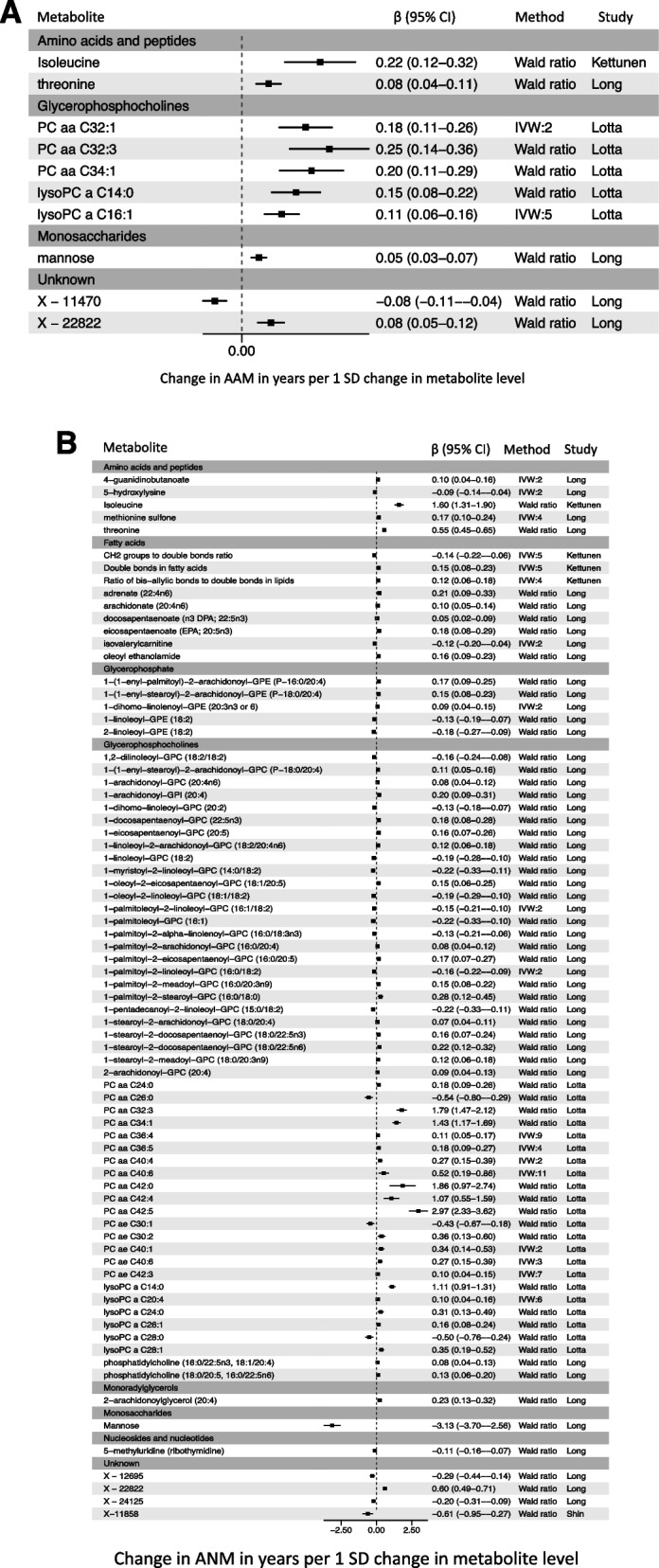
MR-prioritized metabolites for AAM (**A**) or ANM (**B**). Estimates (betas) express changes in years in AAM and ANM per SD increase in the circulating level of each metabolite. The results are grouped based on the main class of the metabolites

Our MR findings indicate causal relationships between ten circulating metabolites and AAM and 76 metabolites for ANM (at an FDR *P*-value ≤ 0.05) (Fig. 2). Among the identified metabolites for AAM, five metabolites belong to the glycerophosphocholine main class, two to the amino acids/peptides, and one to alcohols/polyols. All these metabolites, except X-11470, conferred an increase in AAM (Fig. 2A, Additional file 1: Table S3A), with effects ranging from 0.05 (mannose) to 0.25 (PC aa C32:3) years per 1 SD change in metabolite.

Contrarily, for ANM, metabolites within the same main class exhibited effects in different directions. The most prevalent main class was also the glycerophosphocholines, comprising of 50 of the 76 metabolites, followed by fatty acids with nine metabolites (Fig. 2B, Additional file 1: Table S3B). For ANM, we observed several metabolites, mostly phosphatidylcholine (PC) with absolute MR beta coefficients ranging between 0.05 (docosapentaenoate [n3 DPA; 22:5n3]) and 3.13 (mannose) years per SD increase in the metabolite level.

As statistical tests to evaluate pleiotropy, we performed MR-Egger, MR-PRESSO, and Cochran’s *Q* statistic. These tests did not suggest the presence of pleiotropy in the detected associations for metabolites with more than one SNP-IV (Additional file 1: Tables S4Ai and S4Bi). Additionally, the results of Steiger filtering supported the presumed direction of the causal association, confirming that the candidate metabolites are likely responsible for the changes in AAM and ANM, rather than the inverse (Additional file 1: Tables S4Aii and S4Bii).

**Fig. 3:**
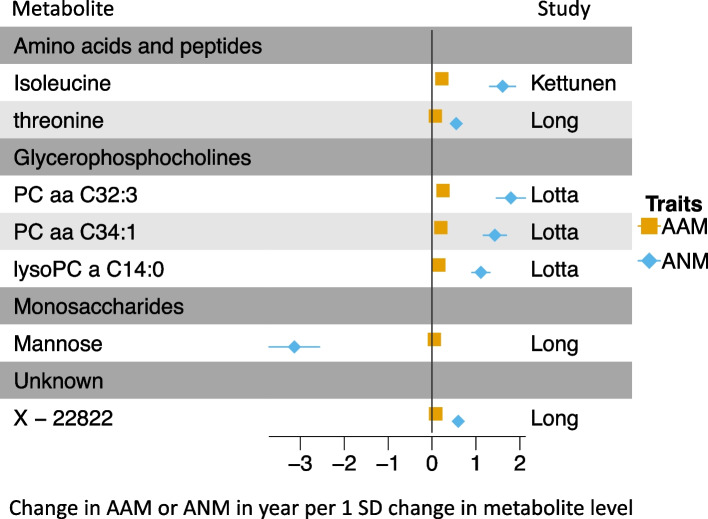
Shared MR-prioritized metabolites between AAM and ANM. Comparison of the effects of shared metabolites between AAM and ANM on the two outcomes. Estimates (betas) express changes in years in AAM and ANM per SD increase in the circulating level of each metabolite. The results are grouped based on the main class of the metabolites

Among the metabolites that met the significance threshold in our MR analyses, seven were common to both AAM and ANM, grouped into four major metabolic clusters: glycerophosphocholines [PC aa C32:3, PC aa C34:1, LysoPC a C14:0], amino acids and peptides [isoleucine, threonine], and monosaccharides [mannoses]. With the exception of mannose for ANM, increasing levels of all the other metabolites were consistently associated with later AAM and later ANM in our MR analyses (Fig. 3 and Additional file 1: Table S5).

### Assessing the influence of BMI on the causal MR associations

To assess the influence of BMI on the causal relationships of the candidate metabolites with AAM or ANM, we conducted MVMR by including either childhood or adult BMI as second exposure in the MR model. These analyses were restricted to MR-prioritized metabolites with three or more SNP-IVs, resulting in one metabolite for AAM and nine for ANM. Among these metabolites, only lysoPC a C20:4 (*P*-value = 0.015), PC aa C36:4 (*P*-value = 0.047), PC aa C40:6 (*P*-value = 0.031), and PC P-40:5 or PC O-40:6 (*P*-value = 0.025) retained a suggestive (*P*-value < 0.05) IVW MR estimate for causal association for ANM after adjusting for adult BMI (Additional file 1: Table S6). The remaining causal associations of metabolites with AAM or ANM disappeared when child or adult BMI was taken into account, suggesting that BMI could potentially mediate or act as a confounder in these associations (Additional file 1: Table S6).

### Colocalization

In our colocalization analyses, we considered that the MR-prioritized metabolites colocalized with AAM or ANM if the posterior probabilities of the candidate metabolites and outcome sharing a single causal SNP (H4) for any of the SNP-IVs of each metabolite were greater than 0.8. We found evidence of colocalization with ANM for 17 MR-prioritized metabolites, mainly from the glycerophosphocholines class, but none for AAM (Additional file 1: Table S7). The genes encompassing the SNP-IVs of the 17 colocalized metabolites were *FADS1*, *FADS2*, *FEN1*, *MYRF*, and *TMEM258*, suggesting the existence of shared pathways among the prioritized metabolites.

### Bidirectional MR analysis

To further validate the directionality of the causal MR associations, we conducted reverse MR, which did not provide evidence for a causal effect of ANM on these metabolites (Additional file 1: Table S8), confirming that the metabolites confer the changes in AAM or ANM, and not the opposite.

### Replication MR analysis

We performed a replication MR analysis utilizing an independent metabolite cohort as a source of IVs for the MR-prioritized metabolites for ANM (Additional file 1: Table S9). The results of the main MR association of omega-3 fatty acids with ANM replicated, with an increase of omega-3 fatty acids levels delaying the onset of ANM, and estimates consistently aligning across various MR methods.

### Metabolic pathway and enrichment analysis

To uncover the biological mechanisms linking the 17 MR-identified and colocalized metabolites with ANM, we conducted a follow-up pathway analysis. Among these metabolites, we were able to identify 15 with Human Metabolome Database (HMDB) identifiers [52], which we used in our metabolite-based pathway and enrichment analysis (Additional file 1: Table S10). Using the KEGG database, we identified a significant association between the glycerophosphocholines cluster and ANM (FDR *P*-value = 1.03 × 10^−9^) (Additional file 1: Table S11A). The pathways underlying this association encompass the metabolism of glycerophospholipids (FDR *P*-value = 2.13 × 10^−3^), alpha-linolenic acid (FDR *P*-value = 2.13 × 10^−3^), and linoleic acid (FDR *P*-value = 8.74 × 10^−3^) (Additional file 1: Table S11B).

**Fig. 4:**
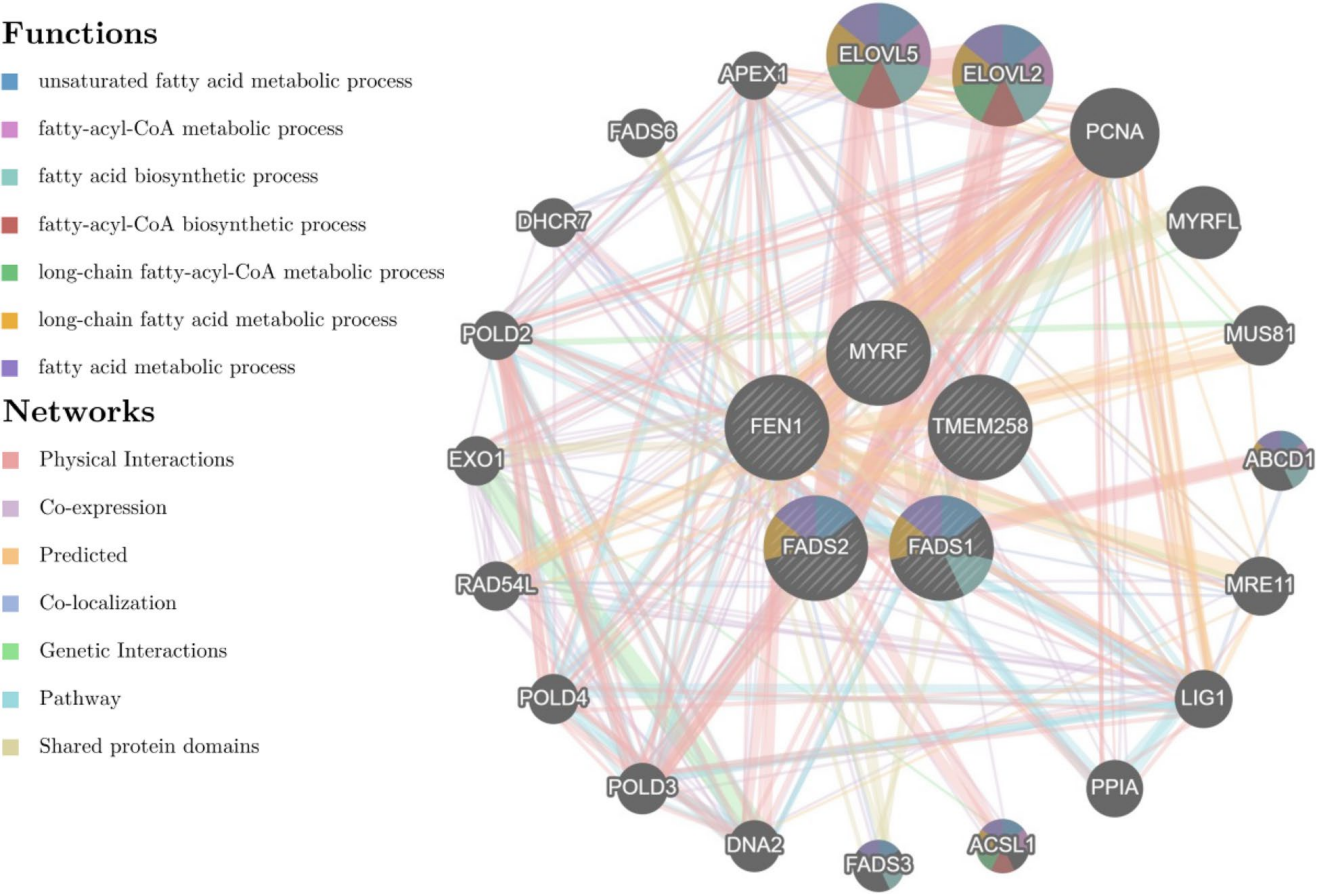
Pathway analysis of colocalized metabolites with ANM using GeneMANIA. Each circle represents a gene and its diverse interactions across the network. Pie-chart for each gene represents specific functions associated with lipid metabolism

The five genes where colocalization between metabolites and ANM occurred shared common networks and functions in our GeneMANIA and FUMA analyses (Fig. 4, Additional file 1: Table S12). Specifically, in our FUMA analysis, both *FADS1* and *FADS2* were linked to the metabolism of linoleic acid (adjusted *P*-value = 9.97 × 10^−4^), alpha-linolenic omega-3, and linoleic omega-6 acids (adjusted *P*-value = 0.001), and to the biosynthesis of unsaturated fatty acids (adjusted *P*-value = 0.003) (Additional file 1: Table S12C). This underlines the importance of the fatty acid pathway in the timing of ANM.

### Validation of selected candidate metabolites for AAM and ANM in ALSPAC

As a further step to validate the MR-prioritized metabolites for AAM and ANM, we conducted an observational study in an independent cohort, ALSPAC. In this cohort, the mean age at menarche was 12.21 ± 1.03 years (*N* = 2456 girls across four visits), and the mean age at menopause was 49.03 ± 4.18 years (*N* = 1626 post-menopausal mothers across the FOM1 and FOM2 visits). Regarding AAM, only two out of the 10 MR-prioritized metabolites were measured in this cohort. However, these two metabolites did not exhibit any association with AAM, with or without adjustment for childhood BMI (Additional file 1: Table S13).

Nine out of the 17 colocalized metabolites for ANM were measured in ALSPAC. Six metabolites were found to be associated (*P*-value < 0.05) with age at menopause in this cohort, all from the fatty acids class. Notably, omega-3 fatty acids displayed the largest effect, with a substantial delay in age at menopause (*β*_*FOM2*_ = 4.12 ± 1.03 years per mmol/l increase in the metabolite level, *P*-value = 6.46 × 10^−5^, *N* = 863). With the exception of monounsaturated fatty acids, all remaining metabolites were consistently associated with a delay in age at menopause, with estimated effects ranging from 0.16 to 4.12 years per unit increase (mmol/l or percent) (Additional file 1: Table S13). After adjusting for BMI, the results remained largely consistent with those from the unadjusted model, except for monounsaturated fatty acids. This discrepancy suggests that BMI may have mediating or confounding effects on the relationship of this metabolite with age at menopause. Furthermore, the majority of our results remained consistent even after excluding mothers with early menopause, defined as an age of menopause < 45 years [49] (Additional file 1: Table S13). Overall, this analysis supports our finding that fatty acids, mostly those associated with omega-3 metabolism, appear to be important in the timing of age at menopause.

## Discussion

In this study, we employed Mendelian randomization (MR) and colocalization analyses to conduct a thorough investigation into the causal relationships between numerous circulating metabolites and the timing of menarche and menopause. We further validated our findings through an observational study in an independent cohort. Our results offer insights into the impact of metabolism, mostly that of the glycerophosphocholines and fatty acids, on female reproductive longevity, indicating that genetic predisposition to altered levels of circulating blood metabolites can be a risk factor for variations in AAM or ANM.

Our analysis highlights the role of many metabolites associated with the choline fraction, clustering within the phosphatidylcholine (PC) subclass, and of fatty acids, both essential nutriments from the diet [53], in the timing of age at menarche and menopause. Metabolism of both phosphatidylcholines and fatty acids involves the enzyme phosphatidylethanolamine N-methyltransferase (PEMT) [54, 55], which is influenced by various factors, including sex hormone levels such as estrogens [56], suggesting links with the female fertility.

Phosphatidylcholine levels are also altered in physiological states, such as pregnancy and menopause [56, 57], and pathological states of estrogen abundance or deficiency. More precisely, postmenopausal women are more susceptible to choline deficiency due to the decline in their estrogen levels, while pregnant women showed protection against its deficiency [56, 57]. Furthermore, this metabolite subclass has been found to play a role in the regulation of menstrual cycle [58] and has shown protective effects on follicular development and oocyte maturation against an exogenous endocrine disruptor [59]. In our MR analysis, the majority of the metabolites associated with AAM or ANM and shared by both, belong to this subclass, with the majority conferring a delay of both outcomes. This underscores the significance of this class in the female reproductive system. Our results for AAM align with previous MR findings [60], while for ANM, further studies are needed to investigate the potential therapeutic effects of choline, or phosphocholine, supplementation on the timing of menopause.

During menopause, there is a shift in unsaturated fatty acid metabolism, and hormonal replacement therapy has been shown to restore different fatty acid levels in postmenopausal women [61] and in animal models [62]. Additionally, fatty acid levels have been found to impact menopausal symptoms [63]. Previous research supports the administration of omega-3 fatty acids to increase ovarian reserve [64], by potentially delaying the onset of menopause. Our main and replication MR analyses, pathway analysis, and observational study in ALSPAC converge to a delaying effect of omega-3, polyunsaturated, and monounsaturated fatty acids, on age at menopause, suggesting a protective role of polyunsaturated and omega-3 fatty acid supplementation in women at risk of premature menopause. A potential mechanism underlying these associations could be linked to the anti-inflammatory properties of omega-3 fatty acids, by reducing the production of proinflammatory cytokines [65]. Indeed, menopausal transition is linked to an increase in markers of inflammation, particularly in women with early menopause, which may suggest a detrimental effect of inflammation on ovarian longevity [66–68]. Thus, a lifetime exposure to higher levels of fatty acids with anti-inflammatory effects could potentially delay the onset of menopause, a hypothesis that merits to be tested in clinical trials.

The involvement of the *FADS1* and *FADS2* genes in ANM is an intriguing finding. These genes encode enzymes responsible for catalyzing the omega-3 and omega-6 lipid biosynthesis pathways [69]. The *FADS* locus, which is clustered on chromosome 11, exerts pleiotropic effects, mostly on lipid-associated metabolic traits, but recent GWAS evidence has linked it with female fertility [70]. Additionally, this locus has been targeted by natural selection multiple times in human history [53, 69, 71], including in populations with diets rich in meat and fish, which are significant sources of omega fatty acids and choline [53]. The selective pressure found in the European population was suggested to be caused by the diet transition across history [53, 70]. However, it can also be possible that the selective pressure could also involve female fertility, potentially by delaying ANM and as such increasing the female reproductive longevity.

Overall, our findings provide new evidence on the role of lipid metabolism in female reproductive longevity, but the precise biological mechanisms behind our findings remain unclear and further studies need to be done to understand these associations.

Our study has multiple strengths. First, we used MR, a study design allowing for causal inference, by limiting confounding, reverse causation, and other biases common in observational epidemiology. The hypothesis-free design of our study offers a thorough screen for causal relationships between metabolites evaluated by non-targeted metabolomics and AAM or ANM. We conducted a number of sensitivity analyses and a replication MR study using instruments from an independent metabolite GWAS, which largely supported the main findings. Our colocalization analyses followed by pathway and enrichment studies provide further insight into the biological mechanisms underlying variations in ANM. Finally, our validation study, based on direct measurements of candidate metabolites in a cohort of women accurately reporting their AAM and ANM, further supports the role of selected MR-prioritized metabolites in these traits.

There are some considerable limitations in our study. Other than BMI, many factors influence the timing of menarche and menopause [1, 2], among which lifestyle traits such as nutrition and physical activity [72–77], which could potentially confound the identified MR associations. However, information about lifestyle factors is not consistently available across cohorts and less GWAS are available of these traits, limiting our ability to adjust for them in multivariable MR. Also, higher BMI is correlated with lower socio-economic status and poor diet. The results of our observational study in ALSPAC girls can be hampered by the fact that the metabolite measurements were predominantly obtained under non-fasting conditions, which may have influenced our results toward the null [78, 79]. Furthermore, the data collection in ALSPAC spanned nearly a decade, during which lifestyle elements may have changed, also potentially mitigating the effects of metabolites on the two outcomes. Analyzing a restricted time period could reduce this bias but might also lead to a loss of statistical power, emphasizing the need for replication in larger datasets with a shorter time frame. Moreover, the definition of age at menopause used in the ReproGen GWAS [12] differs from the definition of age at menopause used in the ALSPAC. While both definitions relied on self-reported age of menopause, which can be subject to memory bias, in the ReproGen GWAS, additional filtering was applied to isolate cases of natural menopause (see Additional file 1: Table S1). Natural menopause is a term used to differentiate the spontaneous occurrence of menopause due to aging versus menopause induced by exogenous or pathological factors. These differences in definitions could potentially explain variations between our discovery and replication analyses. The inclusion of mothers with possible non-natural menopause and the fact that many mothers did not reach menopause during the most recent available follow-up visit in ALSPAC may have contributed to the lower-than-expected mean age at menopause. The memory bias limitation could be alleviated through replication analyses, even if the definitions of age at menopause differ between the discovery and replication phases. Additionally, metabolite levels were not necessarily measured by the same platforms across the metabolomic GWAS and ALSPAC. Also, there is a partial overlap of samples (from the UK Biobank) in the replication MR between the exposure and outcome GWAS cohorts. However, given the robustness of the association between the IVs and exposures (*F*-statistic > 30), and the consistency in direction between the discovery MR and replication in ALSPAC, it is plausible to infer that bias may not necessarily be the driving force behind the observed association. This variation can also affect the results of our validation analysis in ALSPAC. Furthermore, our two-sample MR analyses can only test linear effects of the metabolite levels on AAM and ANM, and therefore, we cannot exclude non-linear effects (i.e., effects of extremely low or high metabolite levels) on these traits. Finally, our results are based on European GWAS data for both metabolites and AAM and ANM, and as such, they cannot be generalized to non-European populations.

## Conclusion

Using complementary approaches leveraging human genomic and metabolomic data, we were able to identify circulating metabolites potentially influencing reproductive longevity. In keeping with previous research, our findings point to choline-containing phospholipids and fatty acids as molecules that affect the timing of both AAM and ANM. These results support the presence of differences in the metabolic profiles of women with altered pubertal or menopausal timing, while unraveling new biological pathways underpinning the female reproductive aging.

### Supplementary Information

**Additional file 1:** **Tables S1 to S13.**

**Additional file 2.** MR-STROBE checklist.

## Data Availability

The GWAS summary data of the five the metabolomic GWAS were obtained from GWAS catalog (accession date: 19 March 2024: Kettunen et al. GWAS: https://www.ebi.ac.uk/gwas/publications/27005778 [13]; Lotta et al. GWAS: https://www.ebi.ac.uk/gwas/publications/33414548 [14]; Shin et al. GWAS: https://www.ebi.ac.uk/gwas/publications/24816252 [16]). Full summary-level results from Long et al. and Suhre et al. are not available, but summary statistics for significant SNPs can be obtained from the GWAS publications (Long et al. GWAS: https://doi.org/10.1038/ng.3809 [15]; Suhre et al. GWAS: https://doi.org/10.1101/2022.06.12.22276286 [35]). The adult and child BMI summary-level GWAS data were also obtained from GWAS catalog (Yengo et al. GWAS: https://www.ebi.ac.uk/gwas/publications/30124842 [30]; Vogelezang et al. GWAS: https://www.ebi.ac.uk/gwas/publications/33045005 [29]). The GWAS data on the ages at menarche [11] and at natural menopause [12] were obtained from the REPROGEN Consortium site (https://www.reprogen.org/data_download.html). The ALSPAC data were accessed under the project number B4178. Additional file 1 (Additional_file_1.xlsx) includes the Tables S1 to S13. Additional file 2 (Additional_file_2.pdf) includes the MR-STROBE checklist.

## References

[1] Ceylan B, Özerdoğan N (2015). Factors affecting age of onset of menopause and determination of quality of life in menopause. Turk J Obstet Gynecol.

[2] Yermachenko A, Dvornyk V (2014). Nongenetic determinants of age at menarche: a systematic review. Biomed Res Int.

[3] Day FR, Elks CE, Murray A, Ong KK, Perry JR (2015). Puberty timing associated with diabetes, cardiovascular disease and also diverse health outcomes in men and women: the UK Biobank study. Sci Rep.

[4] Elks CE, Ong KK, Scott RA, van der Schouw YT, Brand JS, Wark PA (2013). Age at menarche and type 2 diabetes risk: the EPIC-InterAct study. Diabetes Care.

[5] Prentice P, Viner RM (2013). Pubertal timing and adult obesity and cardiometabolic risk in women and men: a systematic review and meta-analysis. Int J Obes (Lond).

[6] Charalampopoulos D, McLoughlin A, Elks CE, Ong KK (2014). Age at menarche and risks of all-cause and cardiovascular death: a systematic review and meta-analysis. Am J Epidemiol.

[7] Bjelland EK, Hofvind S, Byberg L, Eskild A (2018). The relation of age at menarche with age at natural menopause: a population study of 336 788 women in Norway. Hum Reprod.

[8] Burgess S, Daniel RM, Butterworth AS, Thompson SG (2015). Consortium EP-I Network Mendelian randomization: using genetic variants as instrumental variables to investigate mediation in causal pathways. Int J Epidemiol.

[9] Burgess S, Small DS, Thompson SG (2017). A review of instrumental variable estimators for Mendelian randomization. Stat Methods Med Res.

[10] Burgess S, Foley CN, Zuber V (2018). Inferring causal relationships between risk factors and outcomes from genome-wide association study data. Annu Rev Genomics Hum Genet.

[11] Day FR, Thompson DJ, Helgason H, Chasman DI, Finucane H, Sulem P (2017). Genomic analyses identify hundreds of variants associated with age at menarche and support a role for puberty timing in cancer risk. Nat Genet.

[12] Ruth KS, Day FR, Hussain J, Martinez-Marchal A, Aiken CE, Azad A (2021). Genetic insights into biological mechanisms governing human ovarian ageing. Nature.

[13] Kettunen J, Demirkan A, Wurtz P, Draisma HH, Haller T, Rawal R (2016). Genome-wide study for circulating metabolites identifies 62 loci and reveals novel systemic effects of LPA. Nat Commun.

[14] Lotta LA, Pietzner M, Stewart ID, Wittemans LBL, Li C, Bonelli R (2021). A cross-platform approach identifies genetic regulators of human metabolism and health. Nat Genet.

[15] Long T, Hicks M, Yu HC, Biggs WH, Kirkness EF, Menni C (2017). Whole-genome sequencing identifies common-to-rare variants associated with human blood metabolites. Nat Genet.

[16] Shin SY, Fauman EB, Petersen AK, Krumsiek J, Santos R, Huang J (2014). An atlas of genetic influences on human blood metabolites. Nat Genet.

[17] The Genomes Project C, Auton A, Abecasis GR, Altshuler DM, Durbin RM, Abecasis GR, et al. A global reference for human genetic variation. Nature. 2015;526:68. 10.1038/nature15393 PMC4750478 26432245

[18] Palmer TM, Lawlor DA, Harbord RM, Sheehan NA, Tobias JH, Timpson NJ (2012). Using multiple genetic variants as instrumental variables for modifiable risk factors. Stat Methods Med Res.

[19] Park JH, Wacholder S, Gail MH, Peters U, Jacobs KB, Chanock SJ (2010). Estimation of effect size distribution from genome-wide association studies and implications for future discoveries. Nat Genet.

[20] Yavorska OO, Burgess S (2017). MendelianRandomization: an R package for performing Mendelian randomization analyses using summarized data. Int J Epidemiol.

[21] Bowden J, Davey Smith G, Burgess S (2015). Mendelian randomization with invalid instruments: effect estimation and bias detection through Egger regression. Int J Epidemiol.

[22] Verbanck M, Chen CY, Neale B, Do R (2018). Detection of widespread horizontal pleiotropy in causal relationships inferred from Mendelian randomization between complex traits and diseases. Nat Genet.

[23] Hemani G, Tilling K, Davey SG (2017). Orienting the causal relationship between imprecisely measured traits using GWAS summary data. PLoS Genet.

[24] Hemani G, Bowden J, Davey SG (2018). Evaluating the potential role of pleiotropy in Mendelian randomization studies. Hum Mol Genet.

[25] Farahmand M, Ramezani Tehrani F, Azizi F (2012). Whether age of menarche is influenced by body mass index and lipoproteins profile? a retrospective study. Iran J Reprod Med.

[26] Al-Awadhi N, Al-Kandari N, Al-Hasan T, Almurjan D, Ali S, Al-Taiar A (2013). Age at menarche and its relationship to body mass index among adolescent girls in Kuwait. BMC Public Health.

[27] Zhu D, Chung HF, Pandeya N, Dobson AJ, Kuh D, Crawford SL (2018). Body mass index and age at natural menopause: an international pooled analysis of 11 prospective studies. Eur J Epidemiol.

[28] Moore SC, Matthews CE, Sampson JN, Stolzenberg-Solomon RZ, Zheng W, Cai Q (2014). Human metabolic correlates of body mass index. Metabolomics.

[29] Vogelezang S, Bradfield JP, Ahluwalia TS, Curtin JA, Lakka TA, Grarup N (2020). Novel loci for childhood body mass index and shared heritability with adult cardiometabolic traits. PLoS Genet.

[30] Yengo L, Sidorenko J, Kemper KE, Zheng Z, Wood AR, Weedon MN (2018). Meta-analysis of genome-wide association studies for height and body mass index in approximately 700000 individuals of European ancestry. Hum Mol Genet.

[31] Giambartolomei C, Vukcevic D, Schadt EE, Franke L, Hingorani AD, Wallace C (2014). Bayesian test for colocalisation between pairs of genetic association studies using summary statistics. PLoS Genet.

[32] Wallace C (2020). Eliciting priors and relaxing the single causal variant assumption in colocalisation analyses. PLoS Genet.

[33] Bowden J, Davey Smith G, Haycock PC, Burgess S (2016). Consistent estimation in Mendelian randomization with some invalid instruments using a weighted median estimator. Genet Epidemiol.

[34] Hartwig FP, Davey Smith G, Bowden J (2017). Robust inference in summary data Mendelian randomization via the zero modal pleiotropy assumption. Int J Epidemiol.

[35] Karsten S, Raghad A-I, Aziz B, Tanwir H, Anna H, Nisha S, et al. Lipoprotein profile and metabolic fine-mapping of genetic lipid risk loci. medRxiv. 2022:2022.06.12.22276286.

[36] Myers TA, Chanock SJ, Machiela MJ. LDlinkR: An R Package for rapidly calculating linkage disequilibrium statistics in diverse populations. Front Genet. 2020;11:157. 10.3389/fgene.2020.00157. 10.3389/fgene.2020.00157 PMC7059597 32180801

[37] Kanehisa M, Goto S (2000). KEGG: kyoto encyclopedia of genes and genomes. Nucleic Acids Res.

[38] Kim S, Thiessen PA, Bolton EE, Chen J, Fu G, Gindulyte A (2016). PubChem substance and compound databases. Nucleic Acids Res.

[39] Romero P, Wagg J, Green ML, Kaiser D, Krummenacker M, Karp PD (2005). Computational prediction of human metabolic pathways from the complete human genome. Genome Biol.

[40] Degtyarenko K, de Matos P, Ennis M, Hastings J, Zbinden M, McNaught A, et al. ChEBI: a database and ontology for chemical entities of biological interest. Nucleic Acids Res. 2008;36(Database issue):D344–50. 10.1093/nar/gkm791 PMC2238832 17932057

[41] Booth SC, Weljie AM, Turner RJ (2013). Computational tools for the secondary analysis of metabolomics experiments. Comput Struct Biotechnol J.

[42] Sas KM, Karnovsky A, Michailidis G, Pennathur S (2015). Metabolomics and diabetes: analytical and computational approaches. Diabetes.

[43] Pang Z, Chong J, Zhou G, de Lima Morais DA, L Chang, Barrette M (2021). MetaboAnalyst 5.0: narrowing the gap between raw spectra and functional insights. Nucleic Acids Research.

[44] Smith D, Northstone K, Bowring C, Wells N, Crawford M, Pearson RM (2020). The Avon Longitudinal Study of Parents and Children - a resource for COVID-19 research: Generation 2 questionnaire data capture May-July 2020. Wellcome Open Res.

[45] Northstone K, Lewcock M, Groom A, Boyd A, Macleod J, Timpson N (2019). The Avon Longitudinal Study of Parents and Children (ALSPAC): an update on the enrolled sample of index children in 2019. Wellcome Open Res.

[46] Boyd A, Golding J, Macleod J, Lawlor DA, Fraser A, Henderson J (2013). Cohort Profile: the ‘children of the 90s’–the index offspring of the Avon Longitudinal Study of Parents and Children. Int J Epidemiol.

[47] Fraser A, Macdonald-Wallis C, Tilling K, Boyd A, Golding J, Davey Smith G (2013). Cohort Profile: the Avon Longitudinal Study of Parents and Children: ALSPAC mothers cohort. Int J Epidemiol.

[48] Roberts E, Fraser A, Gunnell D, Joinson C, Mars B (2020). Timing of menarche and self-harm in adolescence and adulthood: a population-based cohort study. Psychol Med.

[49] Shuster LT, Rhodes DJ, Gostout BS, Grossardt BR, Rocca WA (2010). Premature menopause or early menopause: Long-term health consequences. Maturitas.

[50] Harris PA, Taylor R, Thielke R, Payne J, Gonzalez N, Conde JG (2009). Research electronic data capture (REDCap)–a metadata-driven methodology and workflow process for providing translational research informatics support. J Biomed Inform.

[51] Santos Ferreira DL, Williams DM, Kangas AJ, Soininen P, Ala-Korpela M, Smith GD (2017). Association of pre-pregnancy body mass index with offspring metabolic profile: Analyses of 3 European prospective birth cohorts. PLoS Med.

[52] Wishart DS, Tzur D, Knox C, Eisner R, Guo AC, Young N, et al. HMDB: the human metabolome database. Nucleic Acids Research. 2007;35(suppl_1):D521-D6. 10.1093/nar/gkl923 PMC1899095 17202168

[53] Buckley MT, Racimo F, Allentoft ME, Jensen MK, Jonsson A, Huang H (2017). Selection in Europeans on fatty acid desaturases associated with dietary changes. Mol Biol Evol.

[54] Zeisel SH (2006). Choline: critical role during fetal development and dietary requirements in adults. Annu Rev Nutr.

[55] Resseguie M, Song J, Niculescu MD, da Costa KA, Randall TA, Zeisel SH. Phosphatidylethanolamine Nmethyltransferase (PEMT) gene expression is induced by estrogen in human and mouse primary hepatocytes. FASEB J. 2007;21(10):2622-32. 10.1096/fj.07-8227com. 10.1096/fj.07-8227com PMC2430895 17456783

[56] Korsmo HW, Jiang X, Caudill MA. Choline: Exploring the growing science on its benefits for moms and babies. Nutrients. 2019;11(8):1823. 10.3390/nu11081823. 10.3390/nu11081823 PMC6722688 31394787

[57] Fischer LM, da Costa KA, Kwock L, Galanko J, Zeisel SH (2010). Dietary choline requirements of women: effects of estrogen and genetic variation. Am J Clin Nutr.

[58] Draper CF, Duisters K, Weger B, Chakrabarti A, Harms AC, Brennan L (2018). Menstrual cycle rhythmicity: metabolic patterns in healthy women. Sci Rep.

[59] Lai FN, Liu XL, Li N, Zhang RQ, Zhao Y, Feng YZ (2018). Phosphatidylcholine could protect the defect of zearalenone exposure on follicular development and oocyte maturation. Aging (Albany NY).

[60] Cheng TS, Day FR, Perry JRB, Luan J, Langenberg C, Forouhi NG, Wareham NJ, Ong KK. Prepubertal dietary and plasma phospholipid fatty acids related to puberty timing: Longitudinal cohort and mendelian randomization analyses. Nutrients. 2021;13(6):1868. 10.3390/nu13061868. 10.3390/nu13061868 PMC8228200 34070864

[61] Cybulska AM, Skonieczna-Żydecka K, Drozd A, Rachubińska K, Pawlik J, Stachowska E, Jurczak A, Grochans E. Fatty acid profile of postmenopausal women receiving, and not receiving, hormone replacement therapy. Int J Environ Res Public Health. 2019;16(21):4273. 10.3390/ijerph16214273. 10.3390/ijerph16214273 PMC6862544 31689897

[62] Gortan Cappellari G, Losurdo P, Mazzucco S, Panizon E, Jevnicar M, Macaluso L (2013). Treatment with n-3 polyunsaturated fatty acids reverses endothelial dysfunction and oxidative stress in experimental menopause. J Nutr Biochem.

[63] Abshirini M, Siassi F, Koohdani F, Qorbani M, Khosravi S, Aslani Z (2019). Higher intake of dietary n-3 PUFA and lower MUFA are associated with fewer menopausal symptoms. Climacteric.

[64] Lipovac M, Aschauer J, Imhof H, Herrmann C, Sima M, Weiss P (2022). The effect of micronutrient supplementation on serum anti-Mullerian hormone levels: a retrospective pilot study. Gynecol Endocrinol.

[65] Calder PC, Grimble RF (2002). Polyunsaturated fatty acids, inflammation and immunity. Eur J Clin Nutr.

[66] Ağaçayak E, Yaman Görük N, Küsen H, Yaman Tunç S, Başaranoğlu S, İçen MS (2016). Role of inflammation and oxidative stress in the etiology of primary ovarian insufficiency. Turk J Obstet Gynecol.

[67] Yasui T, Maegawa M, Tomita J, Miyatani Y, Yamada M, Uemura H (2007). Changes in serum cytokine concentrations during the menopausal transition. Maturitas.

[68] McCarthy M, Raval AP (2020). The peri-menopause in a woman’s life: a systemic inflammatory phase that enables later neurodegenerative disease. J Neuroinflammation.

[69] Ameur A, Enroth S, Johansson A, Zaboli G, Igl W, Johansson AC (2012). Genetic adaptation of fatty-acid metabolism: a human-specific haplotype increasing the biosynthesis of long-chain omega-3 and omega-6 fatty acids. Am J Hum Genet.

[70] Mathieson I, Day FR, Barban N, Tropf FC, Brazel DM, e QC, et al. Genome-wide analysis identifies genetic effects on reproductive success and ongoing natural selection at the FADS locus. Nat Hum Behav. 2023. 10.1038/s41562-023-01528-6 36864135

[71] Fumagalli M, Moltke I, Grarup N, Racimo F, Bjerregaard P, Jørgensen ME (2015). Greenlandic Inuit show genetic signatures of diet and climate adaptation. Science.

[72] Nagata C, Wada K, Nakamura K, Tamai Y, Tsuji M, Shimizu H (2012). Associations of physical activity and diet with the onset of menopause in Japanese women. Menopause.

[73] Sapre S, Thakur R (2014). Lifestyle and dietary factors determine age at natural menopause. J Midlife Health.

[74] Nguyen NTK, Fan H-Y, Tsai M-C, Tung T-H, Huynh QTV, Huang S-Y (2020). Nutrient intake through childhood and early menarche onset in girls: systematic review and meta-analysis. Nutrients.

[75] Malina RM (1983). Menarche in atheletes: a synthesis and hypothesis. Ann Hum Biol.

[76] Chavarro J, Villamor E, Narváez J, Hoyos A (2004). Socio-demographic predictors of age at menarche in a group of Colombian university women. Ann Hum Biol.

[77] Cheng TS, Brage S, van Sluijs EMF, Ong KK (2023). Pre-pubertal accelerometer-assessed physical activity and timing of puberty in British boys and girls: the Millennium Cohort Study. Int J Epidemiol.

[78] Kondoh H, Teruya T, Yanagida M (2020). Metabolomics of human fasting: new insights about old questions. Open Biol.

[79] Emwas AM, Al-Rifai N, Szczepski K, Alsuhaymi S, Rayyan S, Almahasheer H, et al. You are what you eat: application of metabolomics approaches to advance nutrition research. Foods. 2021;10(6):1249. 10.3390/foods10061249 PMC8229064 34072780

