# Supplementary material for: Metabolome-wide Mendelian randomization for age at menarche and age at natural menopause
Source: Genome Med. 2024 May 28;16:69. doi: 10.1186/s13073-024-01322-7 (PMC11131236; doi:10.1186/s13073-024-01322-7)
Supplement: Supplementary file 2 — Additional file 2. MR-STROBE checklist. [file 13073_2024_1322_MOESM2_ESM.pdf]

STROBE-MR checklist of recommended items to address in reports of Mendelian randomization studies[1 2]

Item No.	Section		Checklist item	Page No.	Relevant text from manuscript
1	**TITLE and ABSTRACT**		Indicate Mendelian randomization (MR) as the study's design in the title and/or the abstract if that is a main purpose of the study	1	Metabolome-wide Mendelian randomization for age at menarche and age at natural menopause
	**INTRODUCTION**				
2	**Background**		Explain the scientific background and rationale for the reported study. What is the exposure? Is a potential causal relationship between exposure and outcome plausible? Justify why MR is a helpful method to address the study question	3-4	Introduction, paragraphs 1-3
3	**Objectives**		State specific objectives clearly, including pre-specified causal hypotheses (if any). State that MR is a method that, under specific assumptions, intends to estimate causal effects	3-4	Introduction, paragraph 2<br>MR assumptions section in Method
	**METHODS**				
4	**Study design and data sources**		Present key elements of the study design early in the article. Consider including a table listing sources of data for all phases of the study. For each data source contributing to the analysis, describe the following:		
		a)	Setting: Describe the study design and the underlying population, if possible. Describe the setting, locations, and relevant dates, including periods of recruitment, exposure, follow-up, and data collection, when available.	Additional File 1: Table S1	Detailed data about each GWAS (definitions, recruitment, etc.) in Additional File 1: Table S1
		b)	Participants: Give the eligibility criteria, and the sources and methods of selection of participants. Report the sample size, and whether any power or sample size calculations were carried out prior to the main analysis	Additional File 1: Table S1	See answer to the point above
		c)	Describe measurement, quality control and selection of genetic variants	5	See Instrumental Variables selection in Method
		d)	For each exposure, outcome, and other relevant variables, describe methods of assessment and diagnostic criteria for diseases	Additional File 1: Table S1	See answer to point a above
		e)	Provide details of ethics committee approval and participant informed consent, if relevant		Not applicable
5	**Assumptions**		Explicitly state the three core IV assumptions for the main analysis (relevance, independence and exclusion restriction) as well assumptions for any additional or sensitivity analysis	4-5	See section *Two sample Mendelian Randomization Assumption* in Method

6	**Statistical methods: main analysis**		Describe statistical methods and statistics used		
		a)	Describe how quantitative variables were handled in the analyses (i.e., scale, units, model)	Additional File 1: Table S1<br>5	The units for exposure are in standard deviation and for outcome in year. We mentioned it when describing GWAS cohort (Additional File 1: Table S1, *Discovery Datasets* in Methods and we mentioned the units when mentioning the results)
		b)	Describe how genetic variants were handled in the analyses and, if applicable, how their weights were selected	Additional File 1: Table S1<br>5	The GWAS sources of the betas of the exposure and outcome appear in Additional File 1: Table S1. Selection of the genetic variants is described in *Instrumental Variables selection* section in Methods
		c)	Describe the MR estimator (e.g. two-stage least squares, Wald ratio) and related statistics. Detail the included covariates and, in case of two-sample MR, whether the same covariate set was used for adjustment in the two samples	5-6<br>Additional File 1: Table S1	See section *Mendelian Randomization analysis* in Method. Information about the covariable is in the Additional File 1: Table S1.
		d)	Explain how missing data were addressed	5	For SNPs that were not available in the outcome GWAS, we identified proxy SNPs with high LD (r^2>0.8) using the SNIPA website. (See section *Instrumental Variables selection* in method)
		e)	If applicable, indicate how multiple testing was addressed	6	We corrected for multiple testing using false discovery rate (FDR) (See section *Mendelian Randomization analysis* in Method).
7	**Assessment of assumptions**		Describe any methods or prior knowledge used to assess the assumptions or justify their validity	6-8	See sections *from Mendelian Randomization analysis, Sensitivity analysis, Multivariable MR Analyses, Colocalization analyses* in Method
8	**Sensitivity analyses and additional analyses**		Describe any sensitivity analyses or additional analyses performed (e.g. comparison of effect estimates from different approaches, independent replication, bias analytic techniques, validation of instruments, simulations)	6-8	See sections *Sensitivity analysis, Multivariable MR Analyses, Colocalization analyses* in Method
9	**Software and pre-registration**				
		a)	Name statistical software and package(s), including version and settings used	6-7	For all the MR analysis, we used 'TwoSampleMR v.0.5.5, and its default parameters.<br>For the colocalization, we used the 'coloc' package with the default parameters.

		b)	State whether the study protocol and details were pre-registered (as well as when and where)		Not applicable
	**RESULTS**				
10	**Descriptive data**				
		a)	Report the numbers of individuals at each stage of included studies and reasons for exclusion. Consider use of a flow diagram	Figure 1 and Additional File 1: Table S1	Information about cohorts is presented in Figure 1 and Additional File 1: Table S1
		b)	Report summary statistics for phenotypic exposure(s), outcome(s), and other relevant variables (e.g. means, SDs, proportions)		Summary statistics for exposure are available in Supplementary files
		c)	If the data sources include meta-analyses of previous studies, provide the assessments of heterogeneity across these studies		Not applicable
		d)	For two-sample MR:<br>i. Provide justification of the similarity of the genetic variant-exposure associations between the exposure and outcome samples<br>ii. Provide information on the number of individuals who overlap between the exposure and outcome studies		All GWAS come from European-descent populations. No overlap between exposure and outcome is expected.
11	**Main results**				
		a)	Report the associations between genetic variant and exposure, and between genetic variant and outcome, preferably on an interpretable scale	11-15<br>Figures 2 and 3 (Legend)	Exposure units are in 1-SD, meanwhile the outcome units are in year. It is mentioned in result when reporting a causal relationship.<br>It is also mentioned in legend of figures 2 and 3.
		b)	Report MR estimates of the relationship between exposure and outcome, and the measures of uncertainty from the MR analysis, on an interpretable scale, such as odds ratio or relative risk per SD difference	Additional File 1: Tables S2, S3, S5, S7	All results are presented in Additional File 1: Tables
		c)	If relevant, consider translating estimates of relative risk into absolute risk for a meaningful time period		Not applicable
		d)	Consider plots to visualize results (e.g. forest plot, scatterplot of associations between genetic variants and outcome versus between genetic variants and exposure)	Figures 2 and 3	

12	**Assessment of assumptions**			
		a) Report the assessment of the validity of the assumptions	Additional File 1: Tables S4, S5, S6, S7	The results of the MR sensitivity analysis treating pleiotropy, colocalization, directionality and heterogeneity are presented in Additional File 1: Tables S4, S5, S6, S7
		b) Report any additional statistics (e.g., assessments of heterogeneity across genetic variants, such as I^2, Q statistic or E-value)	Additional File 1: Table S4	Q statistic is reported in Additional File 1: Table S4
13	**Sensitivity analyses and additional analyses**			See point a) and b) above
		a) Report any sensitivity analyses to assess the robustness of the main results to violations of the assumptions		
		b) Report results from other sensitivity analyses or additional analyses		
		c) Report any assessment of direction of causal relationship (e.g., bidirectional MR)		
		d) When relevant, report and compare with estimates from non-MR analyses		Not applicable
		e) Consider additional plots to visualize results (e.g., leave-one-out analyses)		Not applicable
	**DISCUSSION**			
14	**Key results**	Summarize key results with reference to study objectives	15-16	First paragraph of the Discussion
15	**Limitations**	Discuss limitations of the study, taking into account the validity of the IV assumptions, other sources of potential bias, and imprecision. Discuss both direction and magnitude of any potential bias and any efforts to address them	19-20	See *Limitations* section in the Discussion
16	**Interpretation**			
		a) Meaning: Give a cautious overall interpretation of results in the context of their limitations and in comparison with other studies	16-18 19-20	See sections *Glycerophosphocholine metabolite class and omega fatty acids* and *Limitations* in discussion
		b) Mechanism: Discuss underlying biological mechanisms that could drive a potential causal relationship between the investigated exposure and the outcome, and whether the gene-environment equivalence assumption is reasonable. Use causal language carefully, clarifying that IV estimates may provide causal effects only under certain assumptions	16-18	See paragraphs 2,3,5 from the section *Glycerophosphocholine metabolite class and omega fatty acids* in discussion

		c) Clinical relevance: Discuss whether the results have clinical or public policy relevance, and to what extent they inform effect sizes of possible interventions	20	See Conclusion paragraph
17	**Generalizability**	Discuss the generalizability of the study results (a) to other populations, (b) across other exposure periods/timings, and (c) across other levels of exposure	20	It is not generalizable to non-European populations (*Limitations* section in Discussion)
	**OTHER INFORMATION**			
18	**Funding**	Describe sources of funding and the role of funders in the present study and, if applicable, sources of funding for the databases and original study or studies on which the present study is based	21	See Funding paragraph
19	**Data and data sharing**	Provide the data used to perform all analyses or report where and how the data can be accessed, and reference these sources in the article. Provide the statistical code needed to reproduce the results in the article, or report whether the code is publicly accessible and if so, where	21	See *Data availability* paragraph, and supplementary files for exposure
20	**Conflicts of Interest**	All authors should declare all potential conflicts of interest		The authors declare no conflict of interest

This checklist is copyrighted by the Equator Network under the Creative Commons Attribution 3.0 Unported (CC BY 3.0) license.

1. Skrivankova VW, Richmond RC, Woolf BAR, Yarmolinsky J, Davies NM, Swanson SA, et al. Strengthening the Reporting of Observational Studies in Epidemiology using Mendelian Randomization (STROBE-MR) Statement. JAMA. 2021;under review.

2. Skrivankova VW, Richmond RC, Woolf BAR, Davies NM, Swanson SA, VanderWeele TJ, et al. Strengthening the Reporting of Observational Studies in Epidemiology using Mendelian Randomisation (STROBE-MR): Explanation and Elaboration. BMJ. 2021;375:n2233.
